# Mutant non-coding RNA resource in mouse embryonic stem cells

**DOI:** 10.1242/dmm.047803

**Published:** 2021-02-05

**Authors:** Jens Hansen, Harald von Melchner, Wolfgang Wurst

**Affiliations:** 1 Institute of Developmental Genetics, Helmholtz Zentrum München GmbH, German Research Center for Environmental Health, Ingolstädter Landstr. 1, D-85764 Neuherberg, Germany; 2 Department of Molecular Hematology, University Hospital Frankfurt, Goethe University, D-60590 Frankfurt am Main, Germany; 3 Technische Universität München-Weihenstephan, c/o Helmholtz Zentrum München, Ingolstädter Landstr. 1, D-85764 Neuherberg, Germany; 4 German Center for Neurodegenerative Diseases (DZNE), Site Munich, Feodor-Lynen-Str. 17, D-81377 Munich, Germany; 5 Munich Cluster for Systems Neurology (SyNergy), Adolf-Butenandt-Institut, Ludwig-Maximilians-Universität München, Feodor-Lynen-Str. 17, D-81377 München, Germany

**Keywords:** Long non-coding RNA, Mutation, Gene trap, Mouse, *Mus musculus*, Mutagenesis

## Abstract

Gene trapping is a high-throughput approach that has been used to introduce insertional mutations into the genome of mouse embryonic stem (ES) cells. It is performed with generic gene trap vectors that simultaneously mutate and report the expression of the endogenous gene at the site of insertion and provide a DNA sequence tag for the rapid identification of the disrupted gene. Large-scale international efforts assembled a gene trap library of 566,554 ES cell lines with single gene trap integrations distributed throughout the genome. Here, we re-investigated this unique library and identified mutations in 2202 non-coding RNA (ncRNA) genes, in addition to mutations in 12,078 distinct protein-coding genes. Moreover, we found certain types of gene trap vectors preferentially integrating into genes expressing specific long non-coding RNA (lncRNA) biotypes. Together with all other gene-trapped ES cell lines, lncRNA gene-trapped ES cell lines are readily available for functional *in vitro* and *in vivo* studies.

## Abstract (precis)

**Summary:** Analysis of a large library of mouse embryonic stem cell lines with gene trap insertions revealed mutations in 2202 unique non-coding RNA genes, which will significantly contribute to the functional annotation of non-coding RNA genes.

## INTRODUCTION

The comprehensive annotation of the mouse genome has identified over 21,000 protein-coding genes (PCGs), along with more than 15,000 non-coding RNA (ncRNA) genes. To address their function, platforms for large-scale mutagenesis in embryonic stem (ES) cells have been implemented, with the ultimate goal to convert all mutant ES cell lines into mice for subsequent phenotyping. Using high-throughput gene trapping and targeting, the International Knockout Mouse (IKMC) and International Mouse Phenotyping (IMPC) consortia have created an unprecedented resource comprising mutant ES cell lines harboring mutations in ∼18,500 unique PCGs. Of these, over 5000 have been converted into mice and subjected to high-throughput phenotyping (www.mousephenotype.org) (Bradley et al., 2012; Collins et al., 2007; Kaloff et al., 2016; Lloyd et al., 2020; Rosen et al., 2015; Skarnes et al., 2011, 2004). Moreover, genes thus far inaccessible by targeting or trapping are now being addressed individually using CRISPR/Cas9 technology (Brandl et al., 2015; Wefers et al., 2017).

Unlike gene targeting, gene trap strategies rely on generic vectors capable of simultaneously mutating and reporting gene expression at the insertion site as well as providing a sequence tag for seamless gene identification (Friedel and Soriano, 2010). Multiple gene trap vectors have been developed and used in high-throughput screens to generate large libraries of mutant ES cell lines. The vast majority of the ES cell lines assembled by the international consortia were produced with promoter trap vectors, most of which comprise a promoterless reporter and/or selectable marker gene flanked by a 5′ splice acceptor (SA) and a 3′ polyadenylation (pA) sequence (Table S1). Their integrations into an intron of an expressed gene elicits splicing of upstream exons to the reporter gene, resulting in a fusion transcript terminating at the gene trap's pA site and thus truncating the endogenous transcript (Friedrich and Soriano, 1991; Gossler et al., 1989; Skarnes et al., 1992; Wiles et al., 2000; Wurst et al., 1995; Zambrowicz et al., 2003, 1998). Variants thereof either contain type II transmembrane domains fused to the reporter for trapping secretory pathway genes (De-Zolt et al., 2006) or lack a splice acceptor for trapping exons, in which case the reporter is translated from in-frame read-through fusion transcripts (Hicks et al., 1997; von Melchner et al., 1992). Although in theory the latter vector (also referred to as ‘exon traps’) should be activated exclusively from in-frame integrations into exons, in practice a large proportion of these vectors are activated from integrations into introns by adjacent cryptic splice sites (Osipovich et al., 2004). A significantly lower number of ES cell lines were produced with vectors referred to as ‘polyA traps’, in which the reporter genes are flanked by a 5′ constitutive promoter and a 3′ splice donor site, enabling downstream splicing. PolyA trap integrations into introns are expressed from their exogenous promoter and, therefore, unlike most other gene trap vectors, are activated independently of target gene expression (Ishida and Leder, 1999; Niwa et al., 1993; Salminen et al., 1998; Stanford et al., 2006; Yoshida et al., 1995). In a further application, ES cell lines were also generated with gene trap vectors containing both promoter and polyA trap modules, although selection overwhelmingly relied on the promoter trap cassettes (Zambrowicz et al., 2003). Finally, to enable conditional mutagenesis, a significant proportion of ES cell lines were produced with promoter traps equipped with site-specific recombination systems (Schnütgen, 2006; Schnütgen et al., 2005). Overall 566,554 gene-trapped ES cell lines have been produced by the IKMC and can be accessed via the Mouse Genome Informatics (MGI) website (www.informatics.jax.org) (Ringwald et al., 2011). The database covers gene trap integrations into protein-coding and non-coding genes, including long and small non-coding RNA genes.

Long non-coding RNAs (lncRNAs) are defined by a gene length greater than 200 nucleotides, of which 9072 have been annotated in the Ensembl 83 (genome build GRCm38) database. Based on their position relative to PCGs, lncRNA genes were subdivided by the GENCODE consortium into five major classes: (1) long intergenic non-coding RNAs (lincRNAs) located between two protein-coding genes (*n*=3579); (2) antisense lncRNAs transcribed from the opposite strand of coding genes (*n*=2189); (3) ‘sense overlapping’ lncRNAs transcribed from the same strand of protein coding genes (*n*=23 genes); (4) ‘sense intronic’ lncRNAs transcribed from the introns of coding genes (*n*=253); and (5) ‘bidirectional promoter’ lncRNAs transcribed from the opposite strand within the promoter region of a protein-coding gene (*n*=12) (Frankish et al., 2019; Harrow et al., 2012). In addition, several lncRNA genes of numerically minor significance are distributed between the following biotypes (1) ‘processed transcript’ biotype, defined by noncoding transcripts without an open reading frame, (2) ‘3′ overlapping’, defined as short non-coding transcripts transcribed from the 3′UTR, (3) ‘macro’, defined by unspliced lncRNA of several kb in size; and (4) ‘to-be-experimentally-confirmed’ (TEC), defined by non-spliced polyadenylated transcripts with an open reading frame, which, pending further experimental validation, presumably encode novel proteins (Frankish et al., 2019; Harrow et al., 2012).

As key regulators of global gene expression, lncRNAs are involved in the regulation of nearly all fundamental biological processes, including development, cell cycle, differentiation, pluripotency, apoptosis, autophagy and cell migration (Fritah et al., 2014). Hence, it is not surprising that deregulation of lncRNA expression can lead to a wide spectrum of diseases (Rinn and Chang, 2012). However, only a minority of lncRNAs have been functionally validated thus far in tissue culture experiments and knockout mice (Bond et al., 2009; Gomez et al., 2013; Grote and Herrmann, 2015; Li et al., 2013; Liu et al., 2014; Nakagawa et al., 2014; Oliver et al., 2015; Sauvageau et al., 2013; Zhang et al., 2013). Given their biological significance, a large-scale analysis of individual lncRNA function(s) seems highly desirable. To facilitate this endeavor, we re-analyzed the existing gene trap libraries and identified 31,069 ES cell lines with gene trap insertions in 2202 unique ncRNA genes (Tables S4 and S5). This freely available resource should significantly support the functional lncRNA annotation effort.

## RESULTS

### The international gene trap resource

The MGI web portal provides the largest data set of gene trap sequence tags (GTSTs) from mutant murine ES cells generated worldwide by the consortia, institutions and corporations listed with their respective contributions in Table 1. MGI periodically updates vector integration sites by mapping existing GTSTs to the latest mouse genome sequence build (Ringwald et al., 2011). Presently, the database contains 854,155 GTSTs, of which 566,554 are unique. Systematic in-depth analysis of this database revealed 339,779 GTSTs (60%) corresponding to annotated genes and 226,773 (40%) to intergenic regions. For easy accessibility for the user, gene trap clones for a specific gene can be found in the MGI web portal by gene symbol or identifier. All trapped alleles are listed together with information about the vector, the insertion point, the sequence tags and the available mouse lines. Alternatively, a user can search a specified genomic region for gene trap integrations by using the MGI genome browser displaying the gene trap tracks (see tab ‘Search’ and follow the link ‘Mouse Genome Browsers’).

**Table 1.**
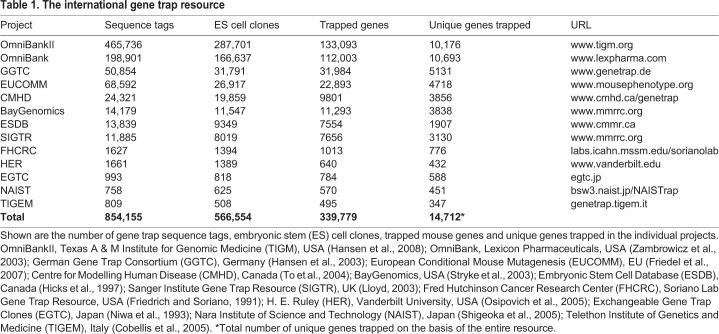
**The international gene trap resource**

### Distribution of gene trap integrations between major gene biotypes

**Fig. 1.**
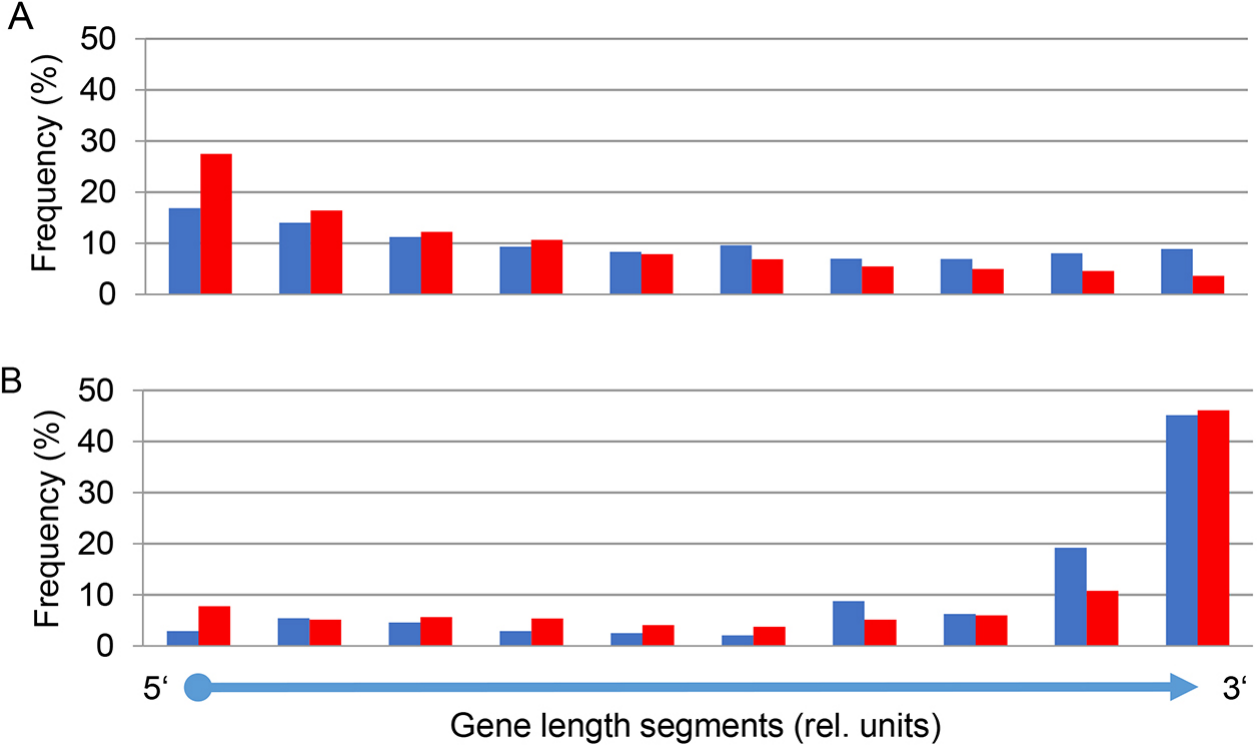
**Integrations of gene trap vectors into gene length segments of long non-coding RNA** **(lncRNA) genes and protein-coding genes (PCGs), measured as distance from the transcription start site.** Each individual gene has been subdivided into ten equal-length segments. lncRNA genes are shown in blue, PCGs in red. (A) Promoter trap vectors with splice acceptor (*n*=196,565). (B) PolyA-trap vectors (*n*=4220). All integrations were analyzed with genomic PCR technologies (see Table S1 for composition of the vector classes). The significance of the integration pattern into gene length segments was studied with a G-test of goodness-of-fit (all *P*-values <10^−16^). rel. units, relative units.

According to their predicted function, the GENCODE consortium (www.gencodegenes.org) subdivides genes into PCGs, lncRNA genes, short non-coding RNA (sncRNA) genes and pseudogenes. Based on this classification, we identified 12,078 (82.1%) of the gene trap integrations in unique PCGs, 2060 (14.0%) in lncRNA genes, 142 (1.0%) in sncRNA genes and 426 (2.9%) in pseudogenes (Table 2). Overall, this corresponds to 55.1% of annotated PCGs and 22.7% of annotated lncRNA genes (Table 2; Tables S4 and S5). Gene trap integrations were significantly enriched in multiple-exon PCGs and processed lncRNA genes consistent with the vast majority of gene trap vectors, for which activation is based on upstream splicing (Tables S2 and S3). Regarding the position of insertion sites relative to transcription start sites, the majority of vectors with SA sites preferred the 5′ ends of both PCGs and lncRNA genes because the larger the 5′ sequence appended to the reporter the less likely the latter will maintain its function. By contrast, polyA trap vectors overwhelmingly select for integration into the 3′ ends of both PCGs and lncRNA genes, as more upstream integrations are generally lost due to nonsense-mediated decay (NMD) (Shigeoka et al., 2005; Stanford et al., 2006) (Fig. 1).

**Table 2.**
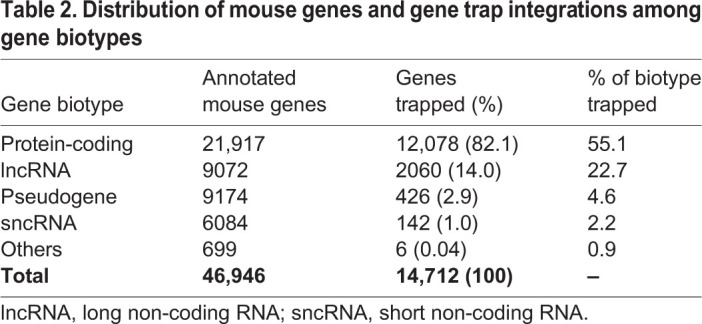
**Distribution of mouse genes and gene trap integrations among gene biotypes**

### Distribution of gene trap insertions between specific ncRNA biotypes

Seventy one percent of the trapped lncRNAs (1455 of 2060) belonged to the lincRNA (806) and antisense RNA (649) biotypes, which together are the most prevalent lncRNAs in the mouse genome (Table 3). Consistent with the general preference of gene traps to mutate larger, multiple-exon genes, only between 1% and 4% of sncRNAs were trapped primarily by vectors lacking SA sites (Table 3). Although in PCGs only ∼0.1% to 0.8% of gene trap insertions occurred in non-spliced genes, insertions into non-spliced lncRNA genes occurred up to 100 times more frequently (1-13%), reflecting the much higher proportion of non-spliced genes among lncRNAs (Table 4; Table S2).

**Table 3.**
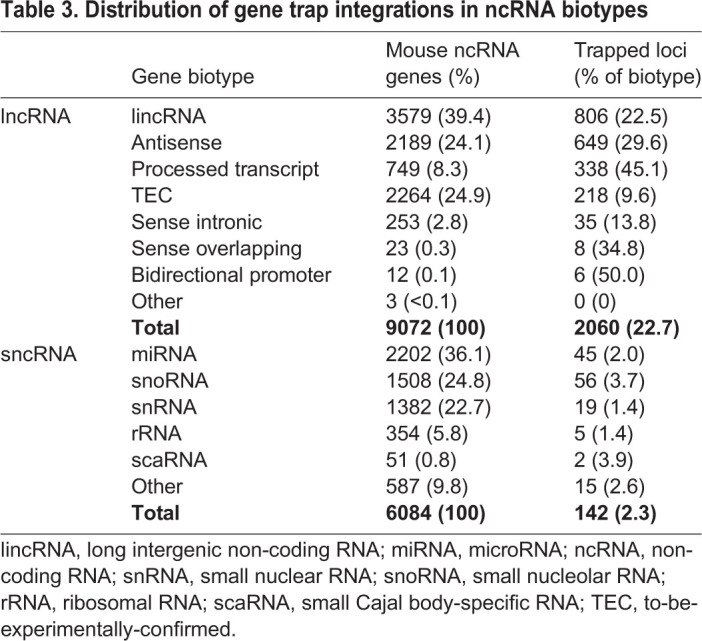
**Distribution of gene trap integrations in ncRNA biotypes**

**Table 4.**
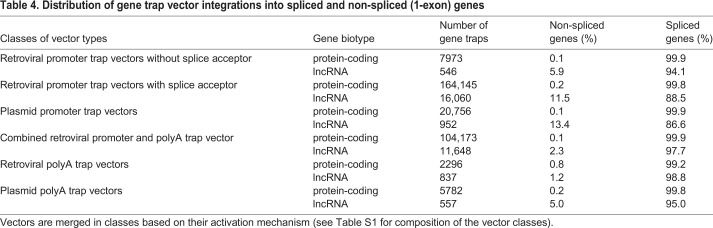
**Distribution of gene trap vector integrations into spliced and non-spliced (1-exon) genes**

**Fig. 2.**
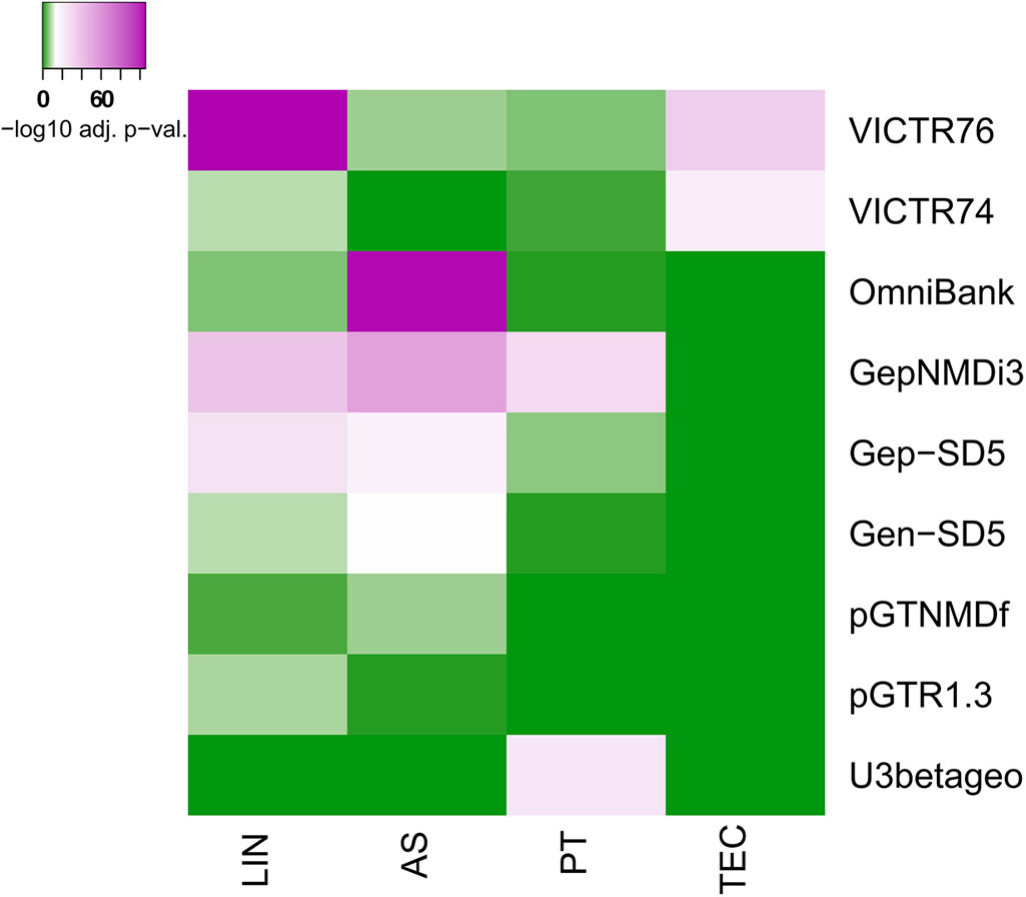
**Heatmap showing the distribution of individual gene trap vector integrations into different gene biotypes.** Colors represent adjusted *P*-values (−log10) of Fisher's exact test for the significant enrichment of gene trap events in at least one gene biotype. *P*-values are corrected for multiple hypothesis testing using the procedure of Benjamini and Hochberg (1995) for false discovery rate estimation. Vectors belong to functional classes as listed in Table S1. AS, antisense; LIN, long intergenic non-coding RNA (lincRNA); PT, processed transcript; TEC, to-be-experimentally-confirmed.

Regarding gene trap integrations into specific lncRNA biotypes, we found a significant relationship between vector type and lncRNA biotype. Fig. 2 shows that the retroviral promoter trap vectors VICTR74 and VICTR76 used for creating the OmniBankII library integrated with much higher frequency into lincRNA and TEC genes than any other similarly structured vectors. Although the reasons for this preference remain unknown, it is likely that the somewhat more sensitive ES cell culture and selection protocols employed for OmniBankII (Hansen et al., 2008) enabled a more efficient isolation of these rather weakly expressed genes (Derrien et al., 2012; Djebali et al., 2012). As TEC genes represent genomic regions presumably encoding novel proteins, the gene trap libraries provide a useful resource for characterizing novel PCGs. Unlike promoter traps, polyA trap vectors, which are activated independently of gene expression, captured lncRNA genes at a much higher rate than any other vectors. For example, the polyA trap vectors GepNMDi3, Gen-SD5, pGTNMDf, pGTR1.3 and Gep-SD5 (To et al., 2004) were all found with high frequency in antisense and lincRNA genes, most of which are either weakly expressed or not expressed at all in ES cells (Ghosal et al., 2013; Jia et al., 2013; Loewer et al., 2010) (Fig. 2).

### Gene trap activation mechanisms in ncRNA genes

**Fig. 3.**
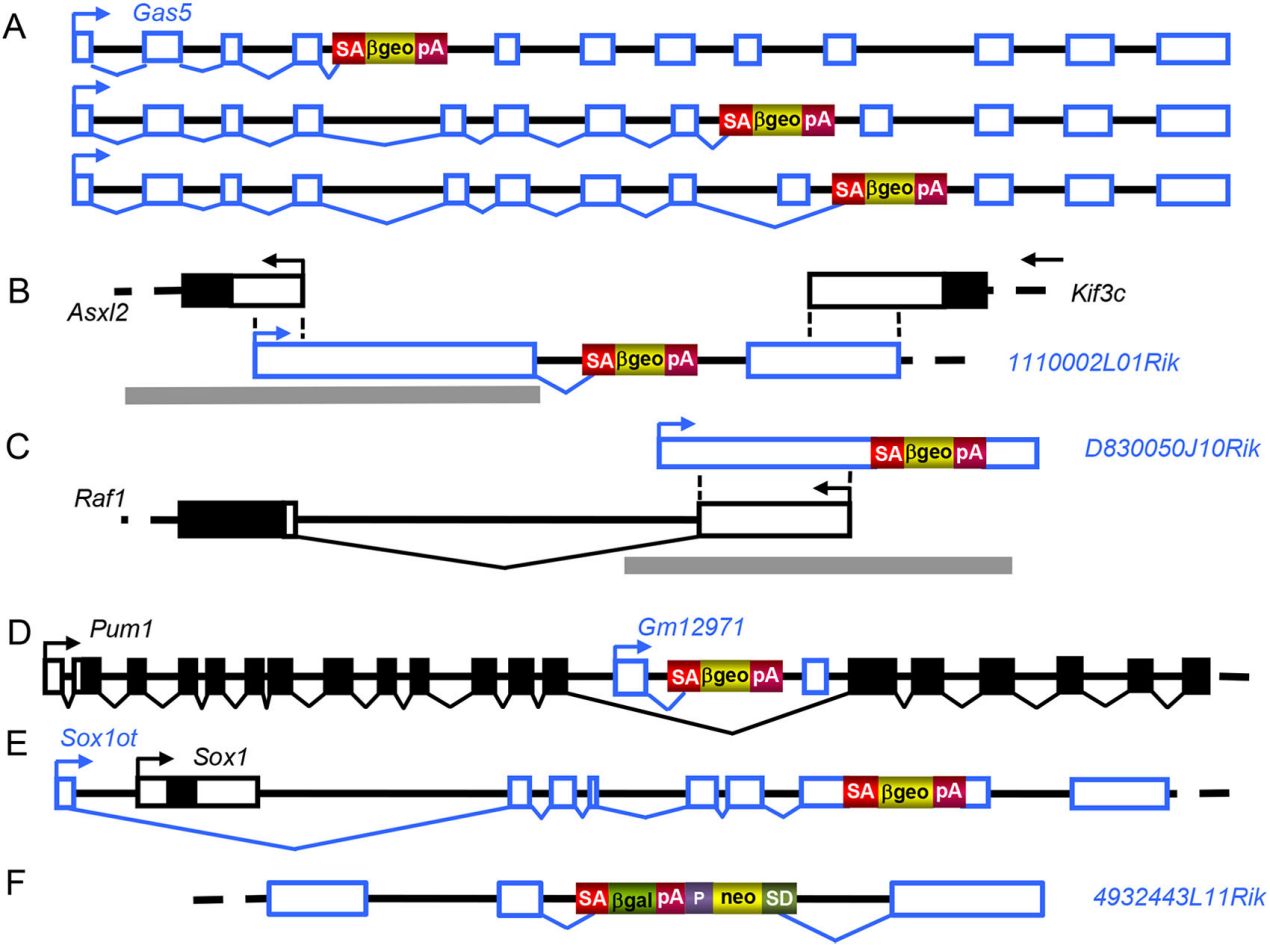
**Gene trap activation from integrations into various lncRNA biotypes.** Exons of lncRNA genes are shown in blue, exons of PCGs in black. Filled bars represent coding sequence, open bars represent non-coding sequence. Transcription start sites and transcriptional orientation are indicated by arrows. Introns are shown as solid black lines, incomplete introns as dashed black lines. Promoters are indicated by thick gray lines. The elements of gene trap selection cassettes are shown in color. βgeo, β-galactosidase-neomycin phosphotransferase fusion gene; βgal, β-galactosidase gene; neo, neomycin phosphotransferase gene; P, promoter; pA, polyA; SA, splice acceptor; SD, splice donor. (A-F) Gene trap vector integrations are shown in the *Gas5* lincRNA gene (A), the antisense *1110002L01Rik* lncRNA gene (B), the bidirectional promoter *D0830050J10Rik* lncRNA gene (C), the sense intronic *Gm12971* lncRNA gene (D), the sense overlapping *Sox1ot* lncRNA gene (E) and the processed transcript *4932443L11Rik* lncRNA gene (F). For further explanations, see text.

Depending on the trapped lncRNA biotype, gene trap integrations were activated by different mechanisms. For example, intron integrations in multiple-exon lincRNAs such as growth arrest-specific transcript 5 (*Gas5*) were activated from the sense strand similar to the activations seen in PCGs (Fig. 3A). By contrast, integrations into the first intron of the *1110002L01Rik* antisense lncRNA, which overlaps the 3′ end of the kinesin family member 3C (*Kif3c*) and the 5′UTR of the additional sex combs-like 2 (*Asxl2*) PCGs, was transcribed in antisense direction to *Asxl2* and *Kif3c* (Fig. 3B). Neither of the PCGs was physically affected by the integration, although mutation of the antisense *1110002L01Rik* transcript could, in principle, interfere with the expression of either gene. A promoter trap integration into the *D0830050J10Rik* bidirectional promoter lncRNA encoded from the opposite strand of the v-raf-leukemia viral oncogene 1 (*Raf1*) PCG was transcribed from the same bidirectional promoter (Fig. 3C), and an integration into the *Gm12971* sense intronic lncRNA was transcribed from its own promoter located in the 14th intron of the *Pum1* PCG (Fig. 3D). Fig. 3E shows an integration into a sense overlapping lncRNA exemplified by Sox1 overlapping transcript (*Sox1ot*), which hosts the SRY (sex determining region Y)-box 1 (*Sox1*) PCG in the first intron. In this arrangement, the fusion transcript initiating at the *Sox1ot* promoter terminates at the gene trap pA site residing in the seventh *Sox1ot* exon (Fig. 3E). Finally, Fig. 3F shows a polyA trap activation from an integration into the last intron of the *4932443L11Rik* processed transcript lncRNA gene by including the gene trap as a portable exon.

## DISCUSSION

In this study, we re-analyzed a library of 566,554 mutant mouse ES cell lines produced in multiple large-scale gene trap mutagenesis projects. Although the library of mutant ES cell lines was originally produced to study the function of PCGs, the present analysis revealed that the library contains 31,069 ES cell lines with mutations in 2202 unique ncRNA genes, in addition to the ES cell lines with mutations in 12,078 unique PCGs, and provides a useful resource for the functional characterization of many ncRNAs. The cell lines can be used *in vitro* to explore the role of ncRNAs in controlling ES cell pluripotency and differentiation (Chakraborty et al., 2012; Dinger et al., 2008; Fisher et al., 2017; Guttman et al., 2011; Sheik Mohamed et al., 2010) and can be readily converted into mutant mice for functional studies at organismal level. It is also worthwhile noting that all traps contain a LacZ reporter, easily enabling the *in vivo* analysis of lncRNA activity at cellular level, which is particularly useful in mutant mouse embryo phenotyping (Dickinson et al., 2016).

The GENCODE reference human and mouse genome annotation database contains three major functional categories of genes: PCGs, non-coding genes and pseudogenes (Harrow et al., 2012). Although gene trap insertions have been found in all these gene classes, a significant proportion involved intergenic regions (Table 2). Considering that 75% of the human genome is covered by primary transcripts and 62% by processed transcripts (Djebali et al., 2012), it is not surprising that 40% of all gene trap integrations were activated from non-annotated genomic regions, thus reflecting the high untapped potential of the gene trap approach for novel gene discovery. In line with this, the existing gene trap resource provides a unique means for resolving the biological significance of not yet annotated genes (Chi, 2016).

Comparison of the integration targets of the different types of gene trap vectors revealed that, owing to fusion transcript size constrictions, promoter trap vectors preferentially integrated near the 5′ ends of both PCGs and multiple-exon lncRNAs (Fig. 1A). However, polyA trap vectors overwhelmingly inserted near the 3′ ends of PCGs and lncRNA genes to produce relatively short fusion transcripts unsusceptible to NMD (Fig. 1B) (Shigeoka et al., 2005; Stanford et al., 2006). In confirmation of previous observations suggesting that gene expression is an important trappability-defining factor for both promoterless and polyA trap vectors (Nord et al., 2007), we found that 90% of the lncRNA genes trapped with promoterless or polyA trap vectors are expressed in ES cells (data not shown).

As ∼900 lncRNAs harbored multiple gene trap integrations at different locations, the ES cell library also provides allelic series for a multitude of lncRNA genes that are extremely useful for specifying distinct functional domains. For example, trapping different regions of the *Gas5* gene resulted in a series of *Gas5* truncation alleles affecting different protein functions (Fig. 3). *Gas5* is a tumor suppressor gene involved in several types of cancer and encodes several molecular functions over its length (Ma et al., 2016), including (1) a glucocorticoid response element (GRE) that competes with DNA for binding to the glucocorticoid receptor DNA-binding domain encoded by a stem-loop structure within the *Gas5* exon 12 (Kino et al., 2010); (2) a mir-21-binding function in exon 4 acting as a miRNA sponge regulating mir-21 levels, which are important in development, cancer, cardiovascular disease and inflammation (Zhang et al., 2013); and (3) an eIF4E-binding function, a key factor of the translation initiation complex (Hu et al., 2014). As shown in Fig. 3A, all these specific functions can be addressed by simply selecting the appropriate gene trap clones for *in vitro* and *in vivo* studies. In support of the *in vivo* value of the lncRNA gene trap lines, Miard et al. (2017) recently published a *Malat1* lncRNA knockout mouse produced with a VICTR74-expressing OmniBankII gene trap clone (IST14461G11). The *Malat1* lncRNA is overexpressed in many types of cancers, including hepatocellular carcinoma, and induces cell proliferation in several cell lines *in vitro*. Although its inactivation had no effect on liver carcinogenesis in mice treated with the genotoxic agent diethylnitrosamine (DEN), DEN-treated knockout mice developed a robust hypercholesterinemia, implicating Malat1 in the regulation of cholesterol metabolism (Miard et al., 2017).

Finally, mutant alleles of lncRNAs containing a reporter gene can nowadays be established *de novo* using CRISPR/Cas9 knock-in strategies in mouse ES cells or mouse zygotes (Wefers et al., 2017; Yao et al., 2018). However, notwithstanding the simplicity of the technology, the generation of allelic series including proper quality controls is still quite time consuming, requiring rigorous genotyping to exclude frequently occurring on-target mutations such as large deletions, insertions, inversions and translocation (Boroviak et al., 2017; Kosicki et al., 2018).

Although the functional characterization of all PCGs is well underway, currently comprising ∼5000 already phenotyped mouse mutants, the next big challenge will be the functional dissection of all non-coding genes for which the existing mutant lncRNA ES cell library provides an unprecedented resource.

## MATERIAL AND METHODS

### Gene trap data

Gene trap sequence tags and their mouse genome coordinates were downloaded from the MGI web portal (www.informatics.jax.org; download on 19 January 2016). We filtered the data set with the objective to finally have one representative sequence tag with a high-quality alignment per vector integration, which was unequivocally mapped to the genome. First, we discarded sequence tags that did not result in a unique high-quality alignment. Insertions that resulted in multiple high-quality alignments and non-successful mappings were also discarded. In a final step all high-quality alignments with the mouse genome, which were indicated as ‘non-representative’, were filtered out.

### Genome data

Software to identify the genomic locus for each gene trap vector insertion site was written in Perl 5.8.8 programming language and uses BioPerl libraries. Genome features at each locus mutated by a gene trap vector integration event were retrieved from the Ensembl database (Yates et al., 2016) using the Ensembl application programming interface (Release 83; www.ensembl.org; genome build GRCm38). Gene models were categorized into biotypes according to the reference gene sets for the mouse published by the GENCODE consortium (version M8 August 2015) (Harrow et al., 2012).

### Statistical testing

To study the significance of gene trap vector integration frequencies over gene length we used a G-test of goodness-of-fit. To determine whether gene trap insertions with a specific vector are over-represented in a given gene biotype, i.e. more integrations are present in genes of a specific gene biotype than expected by chance, a two-by-two contingency table was constructed and Fisher's exact test was performed. The procedure was repeated for each gene trap vector, and adjusted *P*-values were computed to control the false discovery rate (Benjamini and Hochberg, 1995). Categories with a *P*-value not greater than the corresponding adjusted *P*-value were considered significant. The false discovery rate constraint was set to 0.01. All statistical analyses were performed with R statistical software (R v3.3.1; www.r-project.org), using packages stats, RVAideMemoire, gplots and graphics.

## Supplementary Material

Supplementary information
