# Supplementary material for: Mutant non-coding RNA resource in mouse embryonic stem cells
Source: Dis Model Mech. 2021 Feb 5;14(2):dmm047803. doi: 10.1242/dmm.047803 (PMC7875499; doi:10.1242/dmm.047803)
Supplement: Supplementary information [file dmm-14-047803-s1.pdf]

Table S1: Employed gene trap vectors and their mechanism of activation

Vector class		Vector Name	Ref.	Vector Activation
Retroviral promoter trap vectors with splice acceptor	Conditional	FlipROSAbetageo	(1-4)	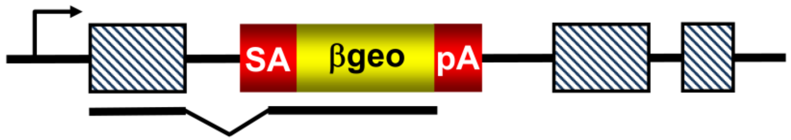
		FlipROSAbgeo0		
		rsFROSAbgeo+2s		
		rsFROSAbgeo0s		
		rsFROSAbgeo+1s		
		eFlpROSAbetageo		
		rFlpROSAbetageo		
		rsFlipROSAbgeo*		
		FlipROSACeoC-2		
		FlipROSACeoC+2		
		rFROSAbgeo+1s		
		rFROSAbgeo+2s		
		rFROSAbgeo0s		
	Non-conditional	ROSAbetageo		
		ROSAbetageo+2		
		ROSAbetageo0		
		VICTR74	(5)	
		VICTR76		
		U3CEO	(6)	
Plasmid promoter trap vectors		pT1betageo	(14,15)	
		pT1ATGbetageo		

Disease Models & Mechanisms • Supplementary information

	pGT0lxr	(7)	
	pGT0Lxf		
	pGT1Lxf		
	pGT2Lxf		
Retroviral promoter trap vectors without splice acceptor	U3neoSV1	(8,9)	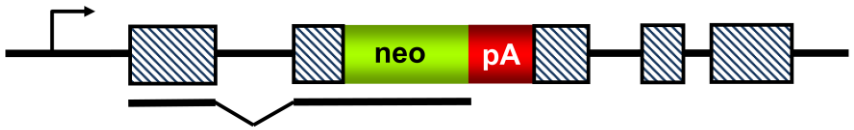
	U3betageo	(10,11)	
Combined retroviral promoter and polyA trap vector	OmniBank	(12)	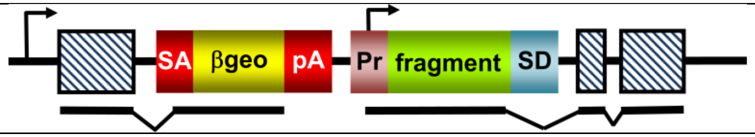
Plasmid polyA-trap vectors	UPA	(13)	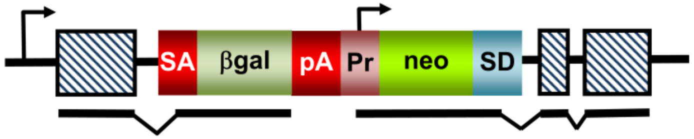
	pGTR1.3		
	pGTR2.3		
	pGTNMDf		
	pGTNMDi		
	pNMDi4		
	pNMDi3		
Retroviral polyA-trap vectors	Gen-SD5	(14)	
	GepNMDi3		
	Gep-SD5		

Disease Models & Mechanisms • Supplementary information

Table S2: Percentage of multiple- and single exon genes annotated in the mouse genome

Gene biotype	Non-spliced genes	Spliced genes
Protein-coding	10.5	89.5
LncRNA	29.5	70.5

Table S3: Distribution of gene trap integrations between different gene biotypes.*

Gene biotype	p-value	odds ratio	adj. p-val.
Protein coding	$<10^{-20}$	10.43649	$<10^{-18}$
LncRNA ‘processed transcript’ subtype	1.16324×10^{-15}	1.820775	2.38464×10^{-14}

*One-sided Fisher’s Exact test. P-values are corrected for multiple hypothesis testing using the procedure of Benjamini & Hochberg (15) for false discovery rate estimation. Gene biotypes with p-value $>10^{-7}$ are not shown.

Table S4: Mouse non-coding RNA genes mutated by gene trap events

Click here to Download Table S4

Table S5: Mutant gene trap alleles in non-coding RNA mouse genes

Click here to Download Table S5

Disease Models & Mechanisms • Supplementary information

References

1. Friedrich, G. and Soriano, P. (1991) Promoter traps in embryonic stem cells: a genetic screen to identify and mutate developmental genes in mice. *Genes Dev.*, **5**, 1513-1523.

2. Schnütgen, F., De-Zolt, S., van Sloun, P., Hollatz, M., Floss, T., Hansen, J., Altschmied, J., Seisenberger, C., Ghyselinck, N.B., Ruiz, P. *et al.* (2005) Genomewide production of multipurpose alleles for the functional analysis of the mouse genome. *Proc. Natl. Acad. Sci. U.S.A.*, **102**, 7221-7226.

3. De-Zolt, S., Schnütgen, F., Seisenberger, C., Hansen, J., Hollatz, M., Floss, T., Ruiz, P., Wurst, W. and von Melchner, H. (2006) High-throughput trapping of secretory pathway genes in mouse embryonic stem cells. *Nucleic Acids Research*, **34**, e25.

4. Schnütgen, F., Hansen, J., De-Zolt, S., Horn, C., Lutz, M., Floss, T., Wurst, W., Noppinger, P.R. and von Melchner, H. (2008) Enhanced gene trapping in mouse embryonic stem cells. *Nucleic Acids Research*, **36**, e133-e133.

5. Hansen, G.M., Markesich, D.C., Burnett, M.B., Zhu, Q., Dionne, K.M., Richter, L.J., Finnell, R.H., Sands, A.T., Zambrowicz, B.P. and Abuin, A. (2008) Large-scale gene trapping in C57BL/6N mouse embryonic stem cells. *Genome Research*, **18**, 1670-1679.

6. Gebauer, M., Melchner, H.V. and Beckers, T. (2001) Genomewide Trapping of Genes that Encode Secreted and Transmembrane Proteins Repressed by Oncogenic Signaling. *Genome Research*, **11**, 1871-1877.

7. Stryke, D., Kawamoto, M., Huang, C.C., Johns, S.J., King, L.A., Harper, C.A., Meng, E.C., Lee, R.E., Yee, A., L'Italien, L. *et al.* (2003) BayGenomics: a resource of insertional mutations in mouse embryonic stem cells. *Nucleic acids research*, **31**, 278-281.

8. Hicks, G.G., Shi, E.-g., Li, X.-M., Li, C.-H., Pawlak, M. and Ruley, H.E. (1997) Functional genomics in mice by tagged sequence mutagenesis. *Nature Genetics*, **16**, 338-344.

9. Osipovich, A.B., Singh, A., Ruley, H.E.E. and Osipovich, A.B. (2005) Post-Entrapment Genome Engineering: First Exon Size does not Affect the Expression of Fusion Transcripts Generated by Gene Entrapment. *Genome Res*, **15**, 428-435.

Disease Models & Mechanisms • Supplementary information

10. Von Melchner, H., DeGregori, J.V., Rayburn, H., Reddy, S., Friedel, C. and Earl Ruley, H. (1992) Selective disruption of genes expressed in totipotent embryonal stem cells. *Genes and Development*, **6**, 919-927.

11. Scherer, C.A., Chen, J., Nachabeh, A., Hopkins, N. and Ruley, H.E. (1996) Transcriptional specificity of the pluripotent embryonic stem cell. *Cell Growth Differ*, **7**, 1393-1401.

12. Zambrowicz, B.P., Abuin, A., Ramirez-Solis, R., Richter, L.J., Piggott, J., BeltrandelRio, H., Buxton, E.C., Edwards, J., Finch, R.A., Friddle, C.J. *et al.* (2003) Wnk1 kinase deficiency lowers blood pressure in mice: A gene-trap screen to identify potential targets for therapeutic intervention. *PNAS*, **100**, 14109-14114.

13. Brickman, J.M., Tsakiridis, A., To, C. and Stanford, W.L. (2010), *Methods in Enzymology*. 2 ed. Elsevier Inc., Vol. 477, pp. 271-295.

14. To, C., Epp, T., Reid, T., Lan, Q., Yu, M., Li, C.Y., Ohishi, M., Hant, P., Tsao, N., Casallo, G. *et al.* (2004) The Centre for Modeling Human Disease Gene Trap resource. *Nucleic Acids Res*, **32**, D557-D559.

15. Benjamini, Y. and Hochberg, Y. (1995), *Journal of the Royal Statistical Society. Series B (Methodological)*, Vol. 57, pp. 289 - 300.

Disease Models & Mechanisms • Supplementary information
